# Isochlorogenic Acid Glucosides from the Arabian Medicinal Plant *Artemisia sieberi* and Their Antimicrobial Activities

**DOI:** 10.3390/molecules28227460

**Published:** 2023-11-07

**Authors:** Khlood Jamal, Areej Al-Taweel, Sarah I. Bukhari, Raha Orfali, Nadine M. S. Moubayed, Jawaher Al-Qahtani, Hanan Aati, Orazio Taglialatela-Scafati, Jiangnan Peng, Shagufta Perveen

**Affiliations:** 1 Department of Pharmacognosy, College of Clinical Pharmacy, Baha University, P.O. Box 26553, Taif 3442, Saudi Arabia; khlood@bu.edu.sa; 2 Department of Pharmacognosy, College of Pharmacy, King Saud University, P.O. Box 22452, Riyadh 11495, Saudi Arabia; amaltaweel@ksu.edu.sa (A.A.-T.); rorfali@ksu.edu.sa (R.O.); jalqahtani@ksu.edu.sa (J.A.-Q.); hati@ksu.edu.sa (H.A.); 3 Department of Pharmaceutics, College of Pharmacy, King Saud University, P.O. Box 22452, Riyadh 11495, Saudi Arabia; sbukhari@ksu.edu.sa; 4 Department of Botany and Microbiology, College of Science, King Saud University, P.O. Box 22452, Riyadh 11495, Saudi Arabia; nmoubayed@ksu.edu.sa; 5 Department of Pharmacy, School of Medicine and Surgery, University of Naples Federico II, Via Montesano 49, 80131 Naples, Italy; scatagli@unina.it; 6 Department of Chemistry, School of Computer, Mathematical and Natural Sciences, Morgan State University, Baltimore, MD 21251, USA; jiangnan.peng@morgan.edu

**Keywords:** *Artemisia sieberi*, Asteraceae, isochlorogenic acids, antifungal activity, antibacterial activity

## Abstract

A phytochemical investigation of the stems of the Arabian plant *Artemisia sieberi* afforded three new isochlorogenic acid derivatives, namely isochlorogenic acid A-3′-*O*-*β*-glucopyranoside (**1**), isochlorogenic acid A-3′-*O*-*β*-glucopyranoside methyl ester (**2**), and isochlorogenic acid C-3′-*O*-*β*-glucopyranoside (**3**), obtained along with thirteen known secondary metabolites belonging to distinct structural classes. The structures of the new metabolites were elucidated by modern spectroscopic techniues based on high-resolution mass spectrometry (HR-ESIMS) and 1D/2D nuclear magnetic resonance (NMR). All isolated compounds were tested for their potential antimicrobial activity against four different bacterial strains (*Bacillus subtilis*, *Staphylococcus aureus*, *Escherichia coli*, and *Pseudomonas aeruginosa*), in addition to a fungal strain (*Candida tropicalis*), The results were expressed as the diameter of the clear zone (in millimetres) around each well. Compounds **1** and **3** (isochlorogenic acid A-3′-*O*-*β*-glucopyranoside and isochlorogenic acid C-3′-*O*-*β*-glucopyranoside, respectively) displayed remarkable antifungal effect and potent antibacterial activities against *B. subtilis* and *S. aureus*, respectively. 3*α*,4*α*-10*β*-trihydroxy-8*α*-acetyloxyguaian-12,6*α*-olide (**6**) and angelicoidenol 2-*O*-*β*-d-glucopyranoside (**9**) emerged as interesting dual antibacterial (selective on *P. aeruginosa*)/antifungal agents.

## 1. Introduction

*Artemisia*, a heterogeneous genus of the Asteraceae family including more than 400 species, is widely distributed in Europe, Asia, South Africa, and North America as a shrub, mug wort, worm wood, and sage brush. The Kingdom of Saudi Arabia is an immense dry country with an area of around 2,250,000 km^2^ that covers a large portion of the Arabian Peninsula and hosts a wide variety of plant species and various ecosystems. Plants of the genus *Artemisia* are represented in Saudi Arabia by seven different species, namely *A. monosperma*, *A. scoparia*, *A. sieberi*, *A. herba-alba*, *A. judaica*, *A. abyssinica* and *A. absinthium*, many of them have found to be used in the folk medicine since ancient times to treat a great variety of ailments [1].

*A. sieberi* is a prominent greenish, perennial shrub that grows in open fields, roadsides, valleys, and desert, with 50–150 cm in height, and pedate, pubescent, alternate leaves. It is widespread in the entire Middle East region [2] as a medicinal plant commonly used as anthelmintic, anti-malarial and for the treatment of gangrenous and infectious ulcers [3]. Several studies have supported the antimicrobial [4], antioxidant [5], hepatoprotective and nephroprotective activities and properties of the extracts. A study reported that the ethanolic extract of *A. sieberi* inhibits angiogenesis both in vitro and in vivo in human umbilical vein endothelial cells and chick chorioallantois membrane models [6].

Although a plethora studies and research work have reported on the composition of the essential oils of this plant, small number of researchers have investigated and explored the phytochemical composition of the plant’s full organic extracts, additionally, none of the activities reported for the extract has been unambiguously associated with a specific metabolite or to a category of compounds. Belonging to the Asteraceae family, it is not surprising that sesquiterpene lactones and flavonoids have been reported as the dominating class of secondary metabolites purified from the medium polarity fractions of the plant extracts. Only a specific and mild antioxidant and antitumor activities have been associated with these compounds [7].

**Figure 1:**
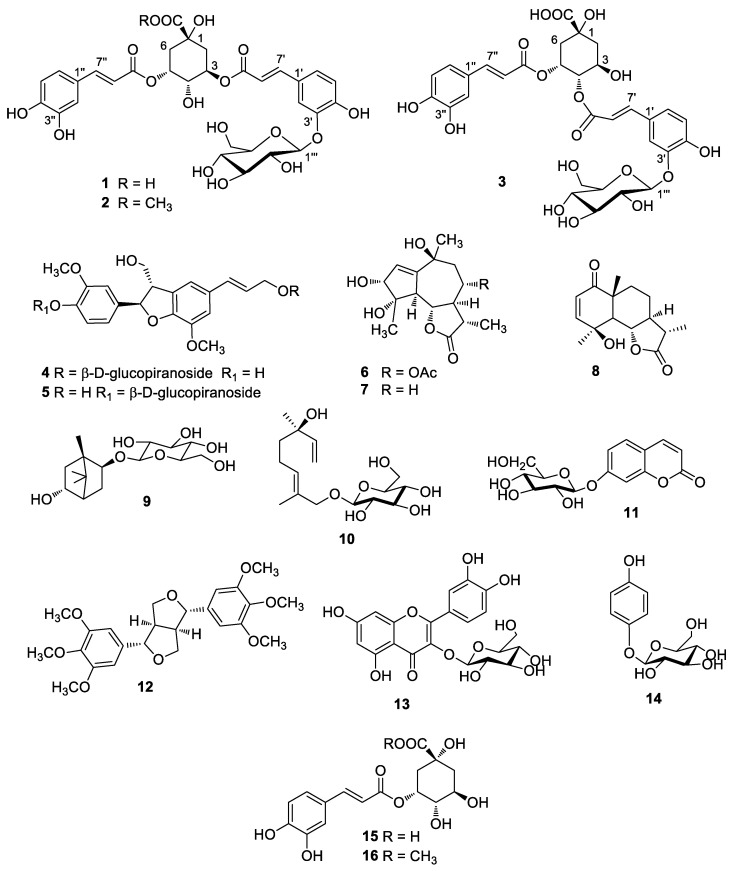
Chemical structures of compounds **1**–**16** isolated from the *A. sieberi*.

Given the need to complete the phytochemical profile of *A. sieberi* with an investigation of the polar fractions of the extract, whose composition is, to our knowledge completely unknown, we were prompted to focus on the phytochemical characterization of this important Saudi *Artemisia* species. Due to the increasing burden of multidrug-resistant strains, there is an urgent worldwide need for new antifungal and antibacterial compounds and agents. To address this issue, we decided to screen the antimicrobial activity and evaluate the biological effects of the purified compounds against five distinct strains of fungi and Gram+ and Gram− bacteria. Our efforts resulted in the characterization of sixteen pure compounds, including three new β-glucopyranoside derivatives of isochlorogenic acids A or C (**1**–**3**, Figure 1), and in the definition of their antimicrobial profile, that gave interesting clues for the development of new antimicrobial agents.

## 2. Results

The Stems part of *A. sieberi* were extracted with a hydroethanolic solution and the extracted gummyish material was macerated in water and then subdivided with dichloromethane followed by *n*-butanol. The non-water soluble parts were chromatographed by using silica gel and then further divided by repeated column and LC fractionations to acquired sixteen compounds (**1**–**16**, Figure 1). These comprised the new compounds isochlorogenic acid A-3′-*O*-*β*-glucopyranoside (**1**), isochlorogenic acid A-3′-*O*-*β*-glucopyranoside methyl ester (**2**), and isochlorogenic acid C-3′-*O*-*β*-glucopyranoside (**3**), and thirteen known secondary metabolites: dehydrodiconiferyl alcohol 9′-*β*-d-glucopyranoside (**4**) [8], dehydrodiconiferyl alcohol 4-*O*-*β*-d-glucopyranoside (**5**) [9], 3*α*,4*α*-10*β*-trihydroxy-8*α*-acetyloxyguaian-12,6*α*-olide (**6**), 3*α*,4*α*-10*β*-trihydroxy-guaian-12,6*α*-olide (**7**), *epi*-*vulgarin* (**8**) [10], angelicoidenol 2-*O*-*β*-d-glucopyranoside (**9**) [11], betulabuside A (**10**) [12], skimmin (**11**), yangambin (**12**) [13], isoquercetin (**13**), *p*-arbutin (**14**), chlorogenic acid (**15**) and its methyl ester (**16**). All the previously known compounds were recognised via correlation of their spectroscopic details with those described in the literature or with those of standards available in our laboratories.

Compound **1** was purified from the butanol soluble fraction as a white powder. The molecular formula of **1** was resloved as C_31_H_34_O_17_ by HR-ESIMS in the negative ion modes (*m*/*z* 677.1781, calcd. 677.1791, for C_31_H_33_O_17_). Diagnostic fragmentation peaks were detected at *m*/*z* 515 and at *m*/*z* 353 and could be compatible with the loss of a caffeoyl and a glycosyl residue, respectively.

The proton NMR spectrum of **1** (Table 1) indicated two sets of mutually coupled doublets (δ_H_ 7.45/6.23 and 7.52/6.37) with coupling constants of 15.5 and 16.1 Hz, respectively, indicative of double bond methines with *trans* configuration, thus supporting the previously hypothesized presence of cinnamyl-type residues. In the aromatic downfield region of the ^1^H NMR spectrum, resonances of two ABX spin systems, at δ_H_ 7.15, 6.98 and 6.77, and at δ_H_ 7.09, 7.14 and 7.12, were suggestive of the existance of two 1,3,4-trisubstituted aromatic rings. Once connected to the directly attached carbon atoms via the 2D NMR HSQC spectrum, these signals could be easily attributed to the resonances of two caffeoyl residues. The remaining signals of the ^1^H NMR spectrum were all located in the middle region and could be only deconvoluted with the help of the 2D NMR COSY spectrum. This revealed the existence of two distinct spin systems: the first included resonances of three different oxymethine protons at δ_H_ 3.62, 5.19 and 5.29, along with two pairs of methylene protons at δ_H_ 1.84 (2H), 1.97 (1H) and 2.15 (1H), in very good agreement with characteristic resonances reported for quinic acid moieties [14,15]; the second spin system started with the doublet at δ_H_ 4.81 (1H), attributale to the anomeric proton of a β-hexopyranose, given its coupling constant of 8.1 Hz. The entire group of ^1^H NMR signals within this spin system, and especially their coupling constants, could be confidently attributed to a β-glucopyranose. All the proton signals of these moieties were connected with the directly linked carbon atoms through HSQC, further supporting the above assignments. The ^13^C NMR spectrum also showed resonances of three carbonyl signals at δ_C_ 177.9, 166.8 and 166.6, six aromatic carbons, including four oxygenated ones, and an unprotonated oxygenated *sp*^3^ carbon resonating at δ_C_ 75.3.

**Figure 2:**
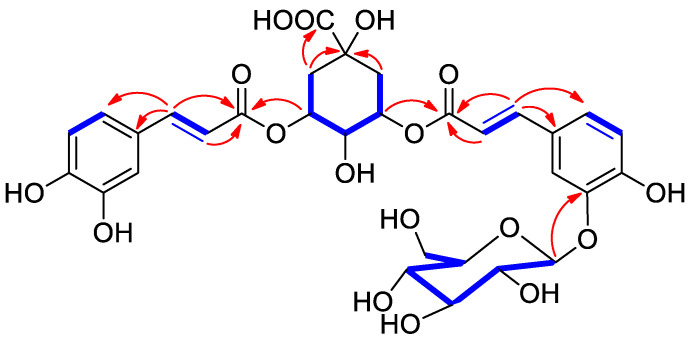
Key ^1^H-^1^H COSY (blue boldened) and HMBC (red arrow) correlations of **1**.

The 2D NMR HMBC spectrum provided crucial ^2,3^*J*_C,H_ connections to finally assemble the above deduced moieties, (Figure 2). Particularly, the cross-peaks of the downfield shifted H-3 (δ_H_ 5.19) and H-5 (δ_H_ 5.29) with C-9′ and C-9″, respectively, indicating the esterification sites of the caffeoyl units on the quinic acid core. The structure of this moiety was further supported by the HMBC cross-peaks of the two methylenes H_2_-2 and H_2_-6 with the carboxylic acid carbonyl carbon. Since several diastereomers of quinic acid have been reported in previously published literature [15], the configuration of quinic acid residue should be assigned with care. A careful comparison of the experimental facts with those reported for chlorogenic acid by Forino et al. [15], allowed a confident assignment of the configuration of this moiety. The point of connectivity of the sugar moiety was deduced by the ^3^*J*_C-H_ HMBC cross-peak detected from the glucosyl anomeric proton at δ_H_ 4.81 to C-3 (δ_C_ 147.7). Acid hydrolysis of compound **1** allowed the identification of the hexose sugar unit as d-glucoside through the optical sign of its rotation.

Consequently, the structure of **1** was characterized as isochlorogenic acid A-3′-*O*-*β*-d-glucopyranoside. The chlorogenic and isochlorogenic acid derivatives represent an important class of natural products, often found in the Asteraceae family, and well known for their bioactivities. Chlorogenic acids have been reported to minimize fasting blood glucose, improve glucose tolerance, enable body weight loss and prevent body weight gain, and promote an improvement in blood pressure in hypertensive patients. In spite of the dozens of chlorogenic acid analogues reported to date, to our updated knowledge, this the first described report of a natural glucosylated derivative of dicaffeoylquinic acid (isochlorogenic acid).

Compound **2** was isolated as white powder with molecular formula C_32_H_36_O_17_, deduced from negative mode HR-ESIMS peak at *m*/*z* 691.1872, (calcd. 691.1880, C_32_H_35_O_17_). ^1^Proton and ^13^Carbon NMR spectra of compound **2** (see Supplementary Materials) were practically superimposable to those of **1**, with the only significant difference of the additional presence of the resonances of a methoxy group (δ_H_ 3.58, δ_C_ 52.4) and the substitutionof the carboxylic group at C-7 with the resonance of an ester carbonyl group (δ_C_ 174.4). The existance of a methyl ester was settled by the ^3^*J* HMBC correlation of δ_H_ 3.58 with carbonyl carbon at δ_C_ 174.4 and its placement secured by the HMBC cross-peaks of H_2-_2 and H_2_-6 with the signal of the ester carbonyl. Thus, in agreement with these spectral features, compound **2** could be assigned as the methyl ester of isochlorogenic acid A-3′-*O*-*β*-glucopyranoside (**1**).

Compound **3** showed the similar molecular formula C_31_H_33_O_17_ as compound **1** and also same ^1^H and ^13^C NMR spectra (Table 1). Analysis of 2D NMR COSY, HSQC, and HMBC of **3** spectra resulted in the allocation of all the carbon and proton resonances and to the explaination of the same moieties above described for **1** (namely two caffeoyl units, a quinic acid and a β-glucopyranose unit). Thus, given the identity of molecular formula and of moieties deduced by spectral analysis, the difference between **1** and **3** must be confined to a different linkage between these moieties. Interestingly, significant differences were found in the pattern of chemical shifts of the quinic acid unit, where the two downfield shifted (as a result of acylation) oxymethines were now vicinal (H-4 and H-5). This location of the acylating caffeoyl units was further supported by the HMBC cross-peaks of H-4 and H-5 with the corresponding ester carbonyl resonances (C-9′ and C-9″, respectively). Having secured the connectivity point of the sugar unit at C-3′ (HMBC cross peak H-1‴/C-3), the structure of **3** can be assigned as isochlorogenic acid C-3′-*O*-*β*-glucopyranoside. This compound could be either a genuine enzymatic product or could be formed as a result of acyl migration on compound **1**, a mechanism not uncommon among acylated polyols.

Thirteen known compounds were also isolated from *A. sieberi*; dehydrodiconiferyl alcohol 9′-*β*-d-glucopyranoside (**4**) [8], dehydrodiconiferyl alcohol 4-*O*-*β*-d-glucopyranoside (**5**) [9], 3*α*,4*α*-10*β*-trihydroxy-8*α*-acetyloxyguaian-12,6*α*-olide (**6**), 3*α*,4*α*-10*β*-trihydroxy-guaian-12,6*α*-olide (**7**), *epi-vulgarin* (**8**) [10], angelicoidenol 2-*O*-*β*-d-glucopyranoside (**9**) [11], betulabuside A (**10**) [12], skimmin (**11**), yangambin (**12**) [13], isoquercetin (**13**), *p*-arbutin (**14**), chlorogenic acid (**15**), and its methyl ester (**16**).

Considering that a significant antibacterial activity must be added to the many showed by chlorogenic acid derivatives [16], in the frame of our current interest in the finding of a new generation of antimicrobial lead compounds from plants origin, we decided to evaluate some of the isolated metabolites (excluding those available in insufficient amounts) on a panel of Gram-+, including *S. aureus*, *B. subtilis* and Gram−, including *E. coli* and *P. aeruginosa*, bacterial strains, in addition to a fungal strain *C. tropicalis*, which are responsible for human diseases in tropical countries [17]. *C. tropicalis* has been indicated as the wide spread pathogenic yeast species after *C. albicans*. Infections due to *C. tropicalis* have increased dramatically around the world thus declare this organism to be an emerging pathogenic yeast. In general, these strains were also chosen for several reasons (a) the relative ease with which they can be propagated and studied in the laboratory; (b) their easy and fast growth; and (c) their susceptibility to several microbial inhibitors at various concentrations. In addition, our choice includes pathogenic and non-pathogenic microorganisms.

The antimicrobial activity of *A. sieberi* extracts and compounds was quantitatively and qualitatively assessed by determining their microorganism’s growth inhibition using agar well diffusion method. Growth inhibitory activity was determined by measuring the clear zone around each well in millimetres (mm) on Mueller–Hinton agar (MHA) plates against tested bacteria and sabouraud dextrose agar plate for fungus strains. All experiments were prepared in triplicate (Table 2). Results indicated the highest growth inhibition against *B. subtilis* was shown by the new compounds **1** and **3**, but also **5** and **10** showed distinct activities. *P. aeruginosa* showed more sensitivity towards the following compounds **4**, **6** and **9**. We can hypothesize that the relatively lower polarity of these compounds can influence in a significant manner this result. Interestingly, compounds **6** and **9** were also the most active against *C. tropicalis* with an almost complete inhibition zone observed. Therefore 3*α*,4*α*-10*β*-trihydroxy-8*α*-acetyloxyguaian-12,6*α*-olide (**6**) and angelicoidenol 2-*O*-*β*-d-glucopyranoside (**9**) qualify as interesting dual antibacterial (selective on Gram negative)/antifungal agents. This detected activity also suggests that the antifungal activity is probably not specific but related to an interaction with a specific, although not identified, target. Indeed, of the two related guaianes **6** and **7**, differing for the presence or absence of an acetoxy group, respectively, only **6** is active. Similarly, of the two related monoterpene glucosides **9** and betulabuside A (**10**), differing for the cyclization of the terpeniyl moiety, only **9** is active. The inhibited activity was noted mainly against *C. tropicalis* with a complete inhibition observed followed by *B. subtilis, S. aureus*, and *P. aeruginosa* and the least effect was recorded with E. coli for the isolated compounds; most compounds have shown antibacterial activity against *P. aeruginosa*.

## 3. Experimental

### 3.1. General Experimental Procedures

An MX-500 Bruker spectrometer was utilized to compute 1D (one-dimensional) and 2D (two-dimensional) NMR (nuclear magnetic resonance) spectra. The NMR δ (chemical shifts) were calculated in ppm compare to TMS, based on the residual signal of the deuterated solvent and *J* scalar coupling constants reported in Hertz (Hz). ESIMS analyses were accomplished on LC/MS Agilent Triple Quadrupole 6410 QQQ mass spectrometer with an ESI ion source (nebulizer gas pressure kept on 60 psi, with 350 °C N_2_ gas temperature having flow rate 12 L/min), operating in the positive and negative mode of scans of ionization through straight infusion method by using MeOH:H_2_O (4:6 *v*/*v*) with a 0.5 mL/ min of flow rate. Purifications and separations of chemical constituents were accomplished by using the open glass column chromatography either RP-18 or silica gel 70–230 mesh (E. Merck, Darmstadt, Germany). The TLC plates used were pre-coated silica gel 60 F254 and RP-18 (Merck), and the visible spots were noticed under UV light and then spraying with ceric sulphate and sulphuric acid reagents and later by heating at 110 °C on a hot plate (TLC plate heater III, CAMAG, Wilmington, NC, USA). Dragendorff’s alkaloid spraying reagent was utilized to give an orange color. Analytical-grade reagent and solvents were purchased from Sigma-Aldrich (St. Louis, MO, USA). Deuterated NMR methanol (CD_3_OD) was purchased from Cambridge Isotope Lab (Tewksbury, MA, USA).

### 3.2. Plant Material

The aerial parts (leaves and stem) of *A. sieberi* were obtained from the city of Jazan of Saudi Arabia, in March 2019 during the blooming stage and recognized by a plant taxonomist, College of Science, King Saud University, dried and immediately extracted. A voucher document specimen (voucher # 16375) has been saved in the herbarium of the Science College, King Saud University, Riyadh (KSU, Saudi Arabia).

### 3.3. Extraction and Isolation

The shade dried powdered leaves and stem parts of *A. sieberi* (leaves 1 kg, and 700 g stems) were ground into powder and extracted by maceration in aqueous ethanol 80% (3 × 2.5 L) till complete exhaustion at room temperature. The crude extract of leaves and stems was filtered and evaporated by using a rotary vacuum evaporator (Buchi, Switzerland) under low pressure at 41 °C to obtain from leaves a dark-green residue (100 g), while the stem a dark brown residue (65 g). The crude extracts were suspended in distilled water and successively extracted by means of liquid–liquid extraction (LLE) with *n*-hexane, chloroform, ethyl acetate, and *n*-butanol and the remaining aqueous fraction were lyophilized. The collective fractions were evaporated by a rotary vacuum evaporator to dryness.

The *n*-BuOH fraction was loaded to Sephadex LH-20 column (3 × 150 cm) eluted with a gradient system of H_2_O:MeOH, starting with 100% water and gradually decreasing the polarity with MeOH up to 100% MeOH. Similar fractions were combined together. Four main fractions were obtained (A–D), which were purified separately on a on silica gel column eluted with a CHCl_3_:MeOH gradient system starting with 0% CHCl_3_ up to 100% methanol. Compounds **7** (3.9 mg), and **8** (0.8 mg) were obtained from the fraction A, compound **9** (3.1 mg) was obtained from fraction B, compounds **11** (0.9 mg) and **12** (0.8 mg), were obtained from fraction C. Compounds **13** (0.9 mg), **14** (0.9 mg), and **15** (4.2 mg) were obtained from fraction D using RP-HPLC.

The EtOAc fraction was loaded to silica gel column and eluted with CHCl_3_:MeOH system to obtain three main fractions (E1-E2-E3) which were re-chromatographed using silica gel column (1.5 × 120 cm) eluted with gradient system of DCM:MeOH H or CHCl_3_:MeOH, starting with 100% DCM/CHCl_3_ and gradient increasing the polarity using MeOH reaching to 100% MeOH, to afford **1** (2.5 mg), **2** (1.8 mg), **3** (2.3 mg), **4** (7.5 mg), **5** (6.8 mg), **6** (5.3 mg), **10** (1.0 mg), and **16** (3.9 mg).

#### 3.3.1. 3-*O*-(3′-*O*-caffeoyl glucosyl)-5-*O*-caffeoylquinic Acid (**1**)

White powder solid. [α]_D_-32 (c 0.1 CD_3_OD). ^1^H and ^13^C NMR: see Table 1. (–) HRESIMS: *m*/*z* = 677.1781 [M − H]^−^. (677.1791 calcd. for C_31_H_33_O_17_).

#### 3.3.2. 3-*O*-(3′-*O*-caffeoyl glucosyl)-5-*O*-caffeoylquinic Acid Methyl Ester (**2**)

White powder solid. [α]_D_-48 (c 0.1 CD_3_OD). (−) HRESIMS: *m*/*z* = 691.1872 [M − H]^–^ (699.1880 calcd. for C_32_H_35_O_17_).

#### 3.3.3. 4-*O*-(3′-*O*-caffeoyl glucosyl)-5-*O*-caffeoylquinic Acid (**3**)

White powder solid. [α]_D_-39 (c 0.1 CD_3_OD). ^1^H and ^13^C NMR: see Table 1. (–) HRESIMS: *m*/*z* = 677.1789 [M − H]^–^. (677.1791 calcd. for C_31_H_33_O_17_).

### 3.4. Antimicrobial Tests

#### 3.4.1. Strains and Growth Condition

All +/− bacterial strains tested in our research and study were kindly provided by the Microbiology Department at King Khaled University Hospital. Four bacterial Strains both gram+ including *B. subtilis* (ATCC 6633) and *S. aureus* (ATCC 29213), and gram—including *E. coli* (ATCC 25966) and *P. aeruginosa* (ATCC 27853), in addition to one fungal strain including *C. tropicalis* (ATCC 66019). All isolates were cultivated on nutrient agar and a bacterial suspension from each isolate of 0.5 McFarland turbidity was produced in 5 mL nutrient broth tubes and Mueller Hinton agar for the antibacterial assays and sabouraud dextrose agar plate for antifungal assay.

#### 3.4.2. Agar Well Diffusion Technique

An antimicrobial evaluation test was performed as described previously. briefly, standard strains were grown for overnight at 37 °C in Nutrient Broth medium. The following day, the suspensions were diluted in Nutrient Broth medium to make This inoculate adjusted to 0.5 McFarland (1 × 10^6^–1 × 10^8^ CFU mL^−1^). Next, each microbial suspension was lay out on the surface of the Mueller Hinton agar (MHA) plates (for bacterial strains) using an alocohol sterile cotton swab or sabouraud dextrose agar plate for fungal strain. MHA surface was pierced with an alocohol sterile cork borer (6 mm) then, 70 µL of each tested compound (amount 5.0 mg/mL in DMSO) was transferred into all well correspondingly. Plates were incubated overnight aerobically for 18–24 h at 37 °C. Diameters of the microbes inhibition zones were measured around all well and the results were recorded as the average of ternary trials in millimeters. Dimethyl sulfoxide (DMSO) was utilized as a negative control while, while ampicillin (25 µg/mL) and fluconazole (50 µg/mL) were the positive control for the bacterial and fungus strains, respectively.

## 4. Conclusions

In conclusion, it is important to discover discover alternatives for antimicrobial agents, especially those of plant origin, and screen these medicinal plants for potential compounds for medicinal and industrial purposes. A phytochemical investigation of the polar fractions obtained from the chromatographic separation of the extract of the Saudi plant *A. sieberi* revealed a complex secondary metabolites chemical profile, including monoterpene glucosides, sesquiterpenes, flavonoid glycosides, lignan, coumarins, etc. The most significant phytochemical result was undoubtedly the isolation and the full characterization of the first glycoside derivatives of isochlorogenic acids A and C (**1**–**3**). Moreover, the acidic derivatives **1** and **3** showed a potent and selective antibacterial activity against *B. subtilis*, while the methyl ester derivative **2** lost this activity showing only a moderate antibacterial action against *S. aureus*. The comparison between these two activity profiles reveals that, while insensitive to the caffeoyl acylation position, the antimicrobial activity is dramatically influenced by the presence of a free (non esterified) carboxylic acid group. Although compounds **1**–**3** were inactive against *C. tropicalis*, the guaianolide derivative **6** and angelicoidenol 2-*O*-*β*-d-glucopyranoside (**9**) showed a potent and previously unreported activity against this fungal strain. Since the same compounds were also active against *P. aeruginosa*, a Gram-negative bacteria responsible for a extensive infections, from respiratory tract infections to skin and urinary tract infections, they qualify as double antibacterial/antifungal lead compounds. This double activity could be extremely beneficial in the treatment of several infections, especially those of the respiratory tract, characterized by co-fungal/bacterial proliferation.

While we are aware that further research is needed to support these preliminary findings, we can confidently state that *A. sieberi* has the potential to produce a large number of metabolites and compounds with antimicrobial properties. It is necessary to conduct additional investigations to determine the effectiveness of these properties in real-world applications against infections and diseases.

## Figures and Tables

**Table 1:** ^1^H NMR (500 MHz) and ^13^C NMR (125 MHz) data for **1** and **3** (in CD_3_OD).

No.	1		3	
	δ_H_ (Mult., *J* in Hz)	δ_c_	δ_H_ (Mult., *J* in Hz)	δ_c_
1		75.3		75.7
2a	1.84 (dd, 12.1, 3.2)	37.8	2.01 (dd, 10.5, 3.2)	37.0
2b	2.15 (d, 12.1, 4.9)		2.09 (dd, 10.5, 5.0)	
3	5.19 (dt, 4.9, 3.2)	73.8	4.30 (bt, 8.5)	68.8
4	3.62 (dd, 8.5, 3.2)	70.5	4.98 (dd, 8.5, 3.2)	75.7
5	5.29 (bt, 8.5)	71.6	5.52 (dt, 4.9, 3.2)	68.0
6a	1.84 (dd, 12.1, 8.5)	39.2	2.07 (dd, 12.1, 8.5)	39.4
6b	1.97 (bd, 12.1)		2.14 (bd, 12.1)	
7		177.9		176.9
1′		129.1		128.8
2′	7.12 (bs)	116.4	7.12 (m)	116.4
3′		147.7		147.7
4′		148.9		148.9
5′	7.09 (d, 7.1)	121.1	7.05 (d, 7.1)	121.9
6′	7.14 (bd, 7.1)	115.0	7.11 (bd, 7.1)	115.4
7′	7.52 (d, 16.1)	144.4	7.53 (d, 15.0)	145.3
8′	6.37 (d, 16.1)	117.4	6.37 (d, 15.0)	116.3
9′		166.8		166.5
1″		125.9		125.8
2″	7.15 (bs)	115.3	7.06 (m)	116.3
3″		146.1		146.1
4″		147.2		147.2
5″	6.98 (d, 7.0)	121.8	6.99 (d, 7.0)	121.4
6″	6.77 (d, 7.0)	116.2	6.77 (d, 7.0)	116.3
7″	7.45 (d, 15.5)	145.3	7.41 (d, 15.0)	145.6
8″	6.23 (d, 15.5)	114.8	6.17 (d, 15.0)	114.3
9″		166.6		166.5
1‴	4.81 (d, 8.1)	101.7	4.79 (d, 8.1)	101.7
2‴	3.31 (m)	73.6	3.30 (m)	73.6
3‴	3.31 (m)	76.2	3.30 (m)	76.2
4‴	3.19 (m)	70.1	3.18 (m)	70.1
5‴	3.36 (m)	77.5	3.35 (m)	77.5
6‴a	3.72 (m)	61.0	3.70 (m)	61.0
6‴b	3.47 (m)		3.47 (m)	

**Table 2:** Antimicrobial activity of test compounds (5.0 mg/mL) expressed as average inhibition zone (in mm).

**Compound**	*S. aureus* ATCC 29213	*B. subtilis* ATCC 6633	*E. coli* ATCC 29213	*P. aeruginosa* ATCC 27853	*C. tropicalis* ATCC 66019
**1**	-	24	-	-	-
**2**	13	-	-	-	-
**3**	-	22	-	-	-
**4**	-	-	-	13	-
**5**	-	23	-	9	9
**6**	-	-	-	13	30
**7**	-	-	-	11	-
**9**	-	-	-	15	30
**10**	-	24	-	-	-
**15**	18	-	-	-	-
**16**	13	-	-	-	-
**Ampicillin**	6	8	8	24	-
**Fluconazole**	-	-	-	-	15

## Data Availability

Data are contained within the article and Supplementary Materials.

## Supplementary Materials

The following supporting information can be downloaded at: https://www.mdpi.com/article/10.3390/molecules28227460/s1, Figure S1: ^1^H NMR spectrum of compound 1 in CD_3_OD. Figure S2: COSY NMR spectrum of compound 1 in CD_3_OD. Figure S3: HSQC NMR spectrum of compound 1 in CD_3_OD. Figure S4: HMBC NMR spectrum of compound 1 in CD_3_OD. Figure S5: ^1^H NMR spectrum of compound 2 in DMSO-d6. Figure S6: ^1^H NMR spectrum of compound 3 in CD_3_OD.

## References

[1] Erel S.B., Reznicek G., Senol S.G., Yavasoglu N.U.K., Konyalioglu S., Zeybek A.U. (2012). Antimicrobial and antioxidant properties of *Artemisia L.* species from western Anatolia. Turk. J. Biol..

[2] Mahboubi M. (2017). *Artemisia sieberi* Besser essential oil and treatment of fungal infections. Biomed. Pharmacother..

[3] Nigam M., Atanassova M., Mishra A.P., Pezzani R., Devkota H.P., Plygun S., Salehi B., Setzer W.N., Sharif-Rad J. (2019). Bioactive compounds and health benefits of Artemisia species. Nat. Prod. Commun..

[4] Mohamed T.A., Hegazy M.E., El Aty A.A., Ghabbour H.A., Alsaid M.S., Shahat A.A., Paré P.W. (2017). Antimicrobial sesquiterpene lactones from *Artemisia sieberi*. J. Asian Nat. Prod. Res..

[5] Mahboubi M., Valian M., Kazempour N. (2015). Chemical composition, antioxidant, and antimicrobial activity of *Artemisia sieberi* oils from different parts of Iran and France. J. Essent. Oil Res..

[6] Abdolmaleki Z., Arab A., Amanpour S., Muhammadnejad S. (2016). Anti-angiogenic effects of ethanolic extract of *Artemisia sieberi* compared to its active substance, artemisinin. Rev. Bras. Farmacogn..

[7] Mohamed T.A., Albadry H.A., Elshamy A.I., Younes S.H.H., Shahat A.A., El-Wassimy M.T., Moustafa M.F., Hegazy M.E. (2021). A new Tetrahydrofuran sesquiterpene skeleton from *Artemisia sieberi*. J. Chin. Chem. Soc..

[8] Adhikari B., Devkota H.P., Joshi K.R., Watanabe T., Yahara S. (2016). Two new diacetylene glycosides: Bhutkesoside A and B from the roots of *Ligusticopsis wallichiana*. Nat. Prod. Res..

[9] Attoumbre J., Hano C., Mesnard F., Lamblin F., Bensaddek L., Raynaud-Le Grandic S., Laine E., Fliniaux M.A., Baltora-Rosset S. (2006). Identification by NMR and accumulation of a neolignan, the dehydrodiconiferyl alcohol-4-β-d-glucoside, in *Linum usitatissimum* cell cultures. C. R. Chim..

[10] Abegaz B., Camps F., Coll J., Feliz M., Jacobsson U., Miravitlles C., Molins E., Torramilans J. (1986). The structures of vulgarin and its isomers: A reinvestigation. Tetrahedron.

[11] Sunnerheim-Sjöberg K. (1992). (1S,2R,4S,5S)-angelicoidenol-2-O-β-D-glucopyranoside. A moose deterrent compound in Scots pine (*Pinus sylvestris* L.). J. Chem. Ecol..

[12] Yue J., Lin Z., Wang D., Sun H. (1994). A sesquiterpene and other constituents from *Erigeron breviscapus*. Phytochem..

[13] Morais L.C.S.L., Almeida R.N., da-Cunha E.V.N., da-Silva M.S., Barbosa-Filho J.M., Gray A.I. (1999). Further lignans from Ocotea duckei. Pharm. Biol..

[14] Flores-Parra A., Guiterrez-Avella D.M., Contreras R., Khuong-Huu F. (1989). 13C and 1H NMR investigations of quinic acid derivatives: Complete spectral assignment and elucidation of preferred conformations. Magn. Res. Chem..

[15] Forino M., Tenore G.C., Tartaglione L., Dell’Aversano C., Novellino E., Ciminiello P. (2015). (1S,3R,4S,5R)5-O-Caffeoylquinic acid: Isolation, stereo-structure characterization and biological activity. Food Chem..

[16] Chen K., Peng C., Chi F., Yu C., Yang Q., Li Z. (2022). Antibacterial and antibiofilm activities of chlorogenic acid against *Yersinia enterocolitica*. Front. Microbiol..

[17] Chai L.Y.A., Denning D.W., Warn P. (2010). *Candida tropicalis* in human disease. Crit. Rev. Microbiol..

