# Supplementary material for: Isochlorogenic Acid Glucosides from the Arabian Medicinal Plant Artemisia sieberi and Their Antimicrobial Activities
Source: Molecules. 2023 Nov 7;28(22):7460. doi: 10.3390/molecules28227460 (PMC10673327; doi:10.3390/molecules28227460)
Supplement: Supplementary file 1 [file molecules-28-07460-s001.zip › molecules-2668042-supplementary.pdf]

SUPPLEMENTARY MATERIAL

Isochlorogenic Acid Glucosides from the Arabian Medicinal Plant *Artemisia sieberi* and Their Antimicrobial Activities

Khlood Jamal[a], Areej M. Al-Taweel,[b] Sarah I. Bukhari,[c] Nadine M.S Moubayed,[d] Jawaher Al-Qahtani,[b] Orazio Taglialatela-Scafati[e] and Shagufta Perveen[f,*]

[a]*Department of Pharmacognosy, College of Clinical Pharmacy, Baha University. P. O. Box 26553, Taif, 8287, Kingdom of Saudi Arabia*

[b]*Department of Pharmacognosy, College of Pharmacy, King Saud University. P. O. Box 22452, Riyadh 11495, Kingdom of Saudi Arabia*

[c]*Department of Pharmaceutics, College of Pharmacy, King Saud University. P. O. Box 22452, Riyadh 11495, Kingdom of Saudi Arabia*

[d]*Deparment of Botany and Microbiology, College of Science, King Saud University, Riyadh 11495, Kingdom of Saudi Arabia*

[e]*Department of Pharmacy, School of Medicine and Surgery, University of Naples Federico II, Via Montesano 49, 80131 Naples, Italy*

[f]*Department of Chemistry, School of Computer, Mathematical and Natural Sciences, Morgan State University, Baltimore, MD 21251, USA*

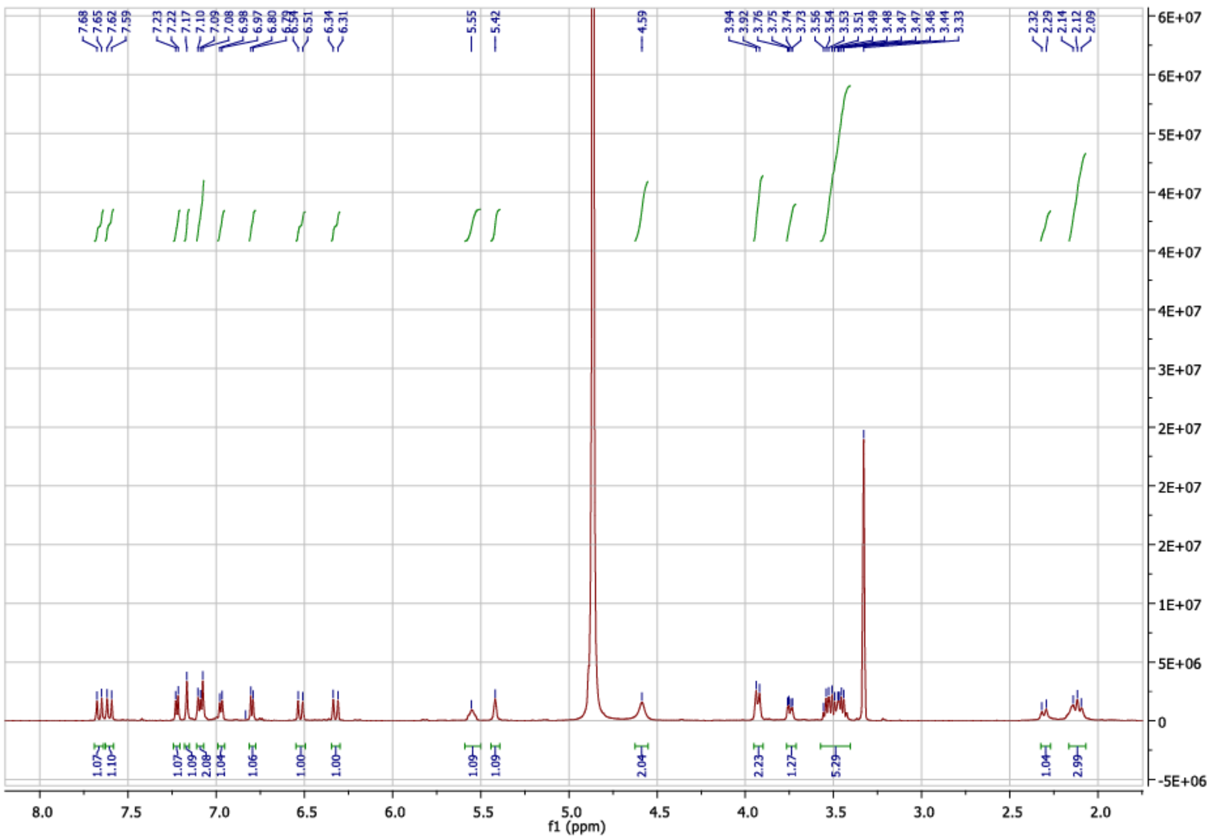

Figure S1. ^{1}H NMR spectrum of compound **1** in CD_3OD

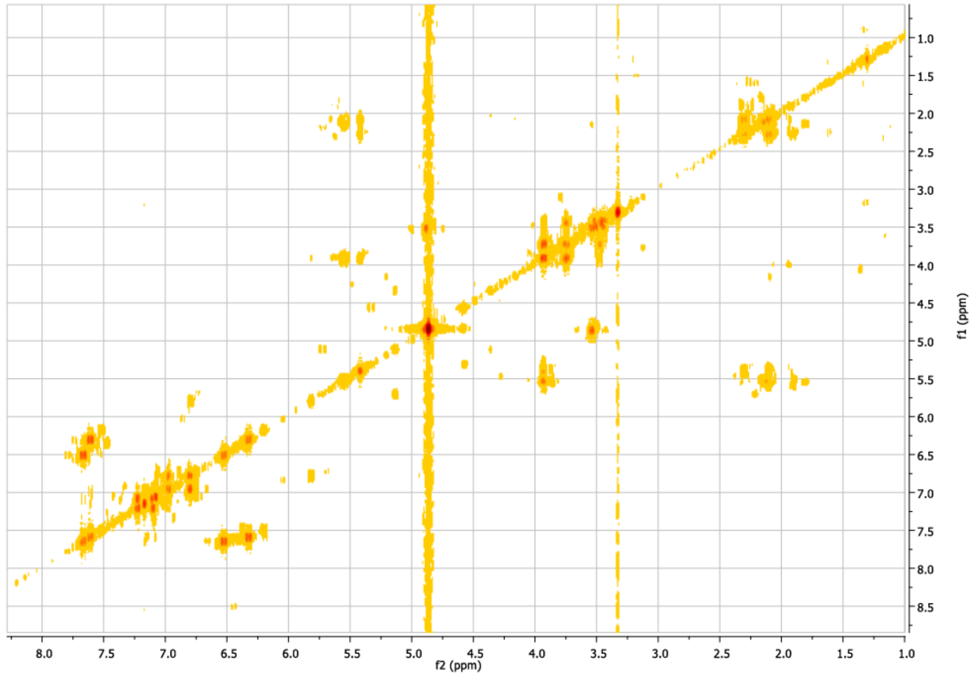

Figure S2. COSY NMR spectrum of compound **1** in CD_3OD

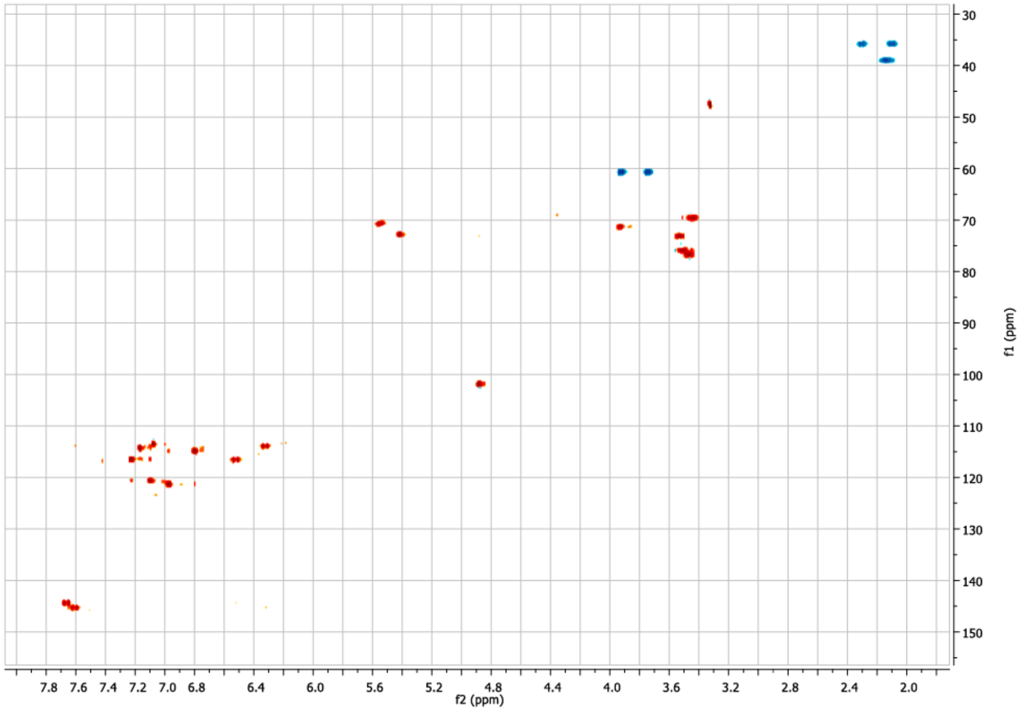

Figure S3. HSQC NMR spectrum of compound **1** in CD_3OD

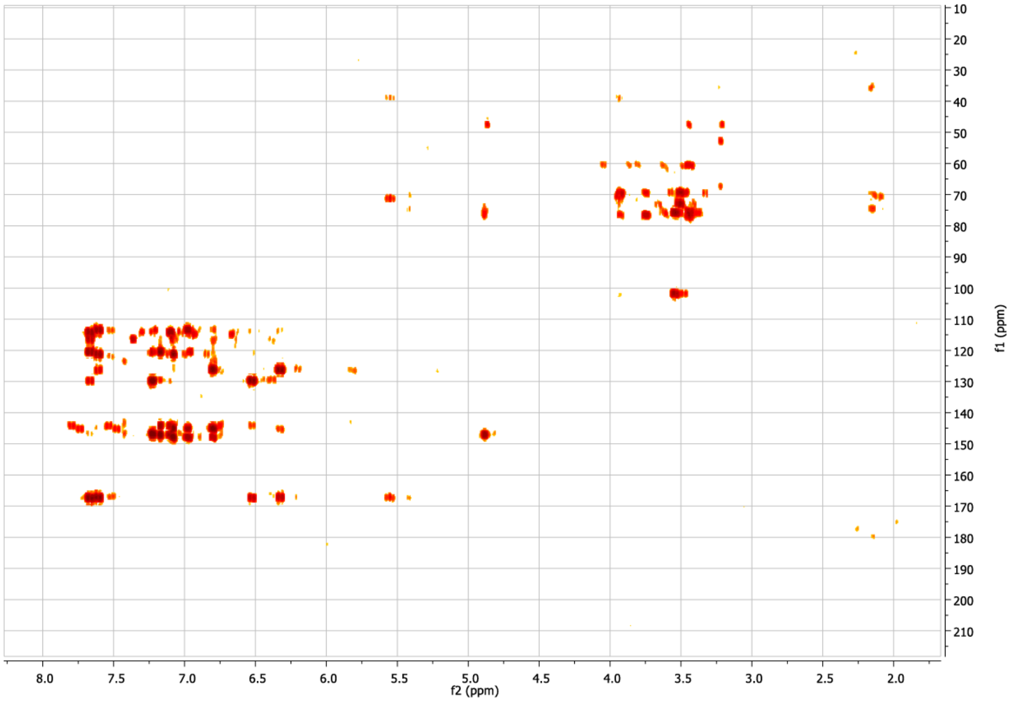

Figure S4. HMBC NMR spectrum of compound **1** in CD_3OD

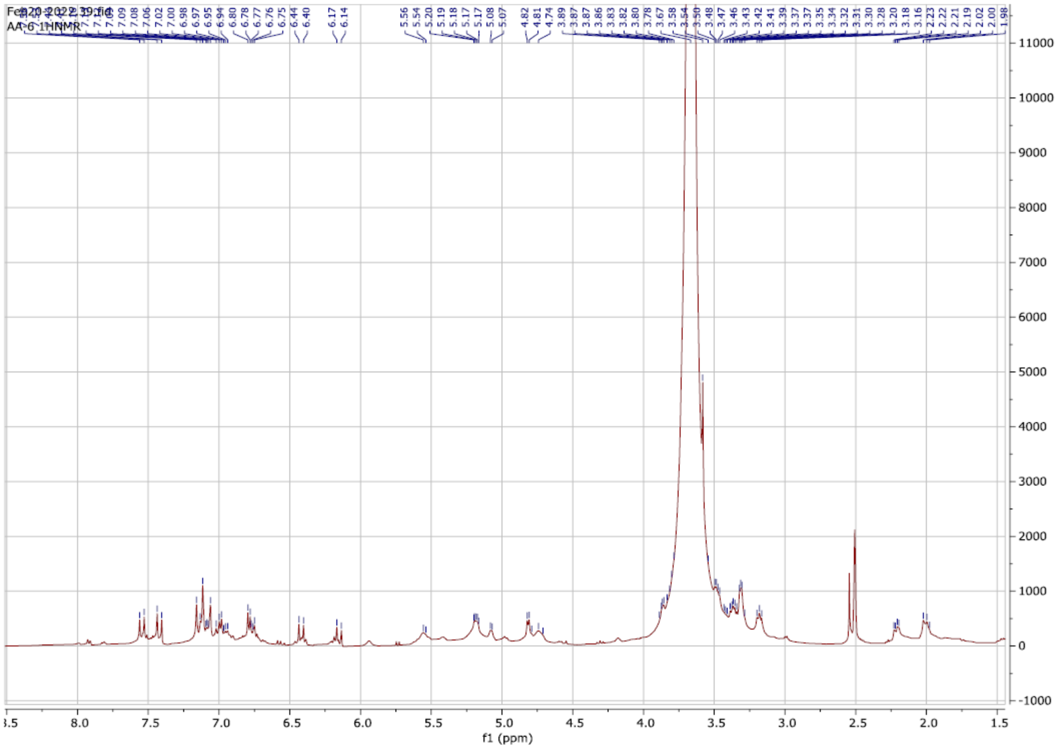

Figure S5. ^{1}H NMR spectrum of compound **2** in DMSO-d6

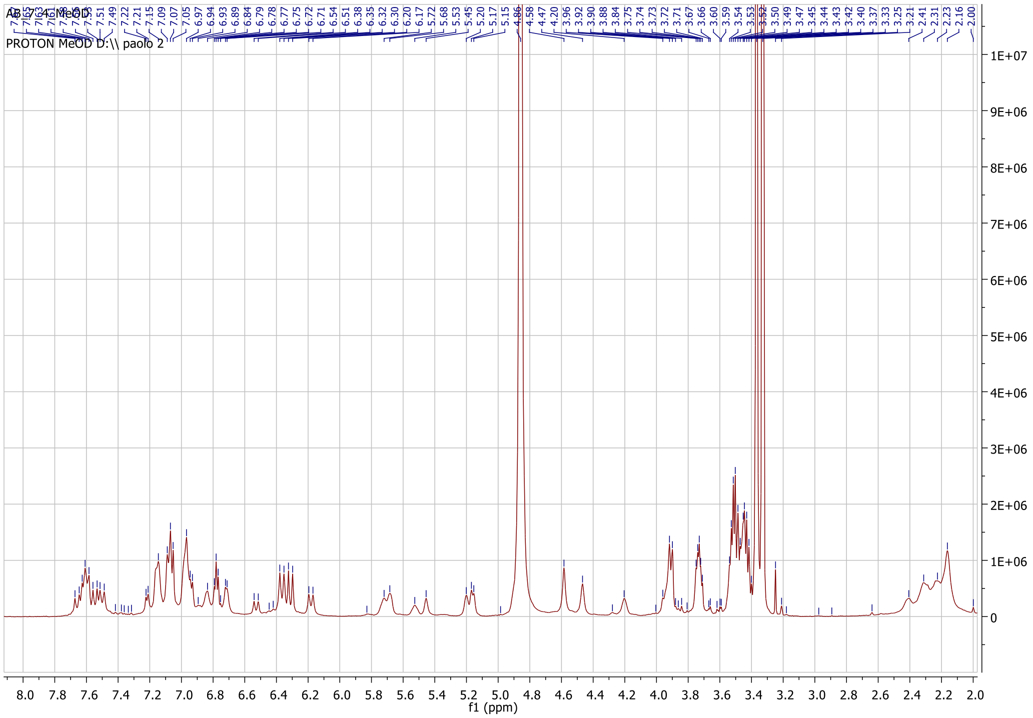

Figure S6. ^{1}H NMR spectrum of compound **3** in CD_3OD
